# Antibacterial activities of almond skins on *cagA*-positive and-negative clinical isolates of *Helicobacter pylori*

**DOI:** 10.1186/1471-2180-13-103

**Published:** 2013-05-09

**Authors:** Carlo Bisignano, Angela Filocamo, Erminia La Camera, Sebastiana Zummo, Maria Teresa Fera, Giuseppina Mandalari

**Affiliations:** 1 Dipartimento di Scienze del Farmaco e Prodotti per la Salute, University of Messina, Viale Annunziata, Messina, 98100, Italy; 2 Dipartimento di Scienze Pediatriche, Ginecologiche, Microbiologiche e Biomediche, University of Messina, Policlinico Universitario, Viale Gazzi, Messina, 98125, Italy; 3 The Model Gut, Institute of Food Research, Norwich Research Park, Colney Lane, Norwich, NR4 7UA, UK

**Keywords:** *Helicobacter pylori*, Flavonoids, Almond skins, Epicathechin, Naringerin, Protocatechuic acid

## Abstract

**Background:**

*Helicobacter pylori* is known to be a gastric pathogen of humans. Eradication regimens for *H. pylori* infection have some side effects, compliance problems, relapses, and antibiotic resistance. Therefore, the need for alternative therapies for *H. pylori* infections is of special interest. We have previously shown that polyphenols from almond skins are active against a range of food-borne pathogens. The aim of this study was to evaluate the antibacterial effects of natural almond skins before and after simulated human digestion and the pure flavonoid compounds epicatechin, naringenin and protocatechuic acid against *H. pylori.*

**Results:**

*H. pylori* strains were isolated from gastric biopsy samples following standard microbiology procedures. Also, *cagA* and *vacA* genes were identified using PCR. Susceptibility studies on 34 strains of *H. pylori*, including two reference strains (ATCC 43504, ATCC 49503), were performed by the standard agar dilution method.

Natural almond skin was the most effective compound against *H. pylori* (MIC range, 64 to 128 μg/ml), followed by natural skin post gastric digestion (MIC range, 128 to 512 μg/ml), and natural almond skin post gastric plus duodenal digestion (MIC range, 256 to 512 μg/ml). Amongst the pure flavonoid compounds, protocatechuic acid showed the greatest activity (MIC range, 128 to 512 μg/ml) against *H. pylori* strains.

**Conclusions:**

Polyphenols from almond skins were effective in vitro against *H. pylori*, irrespective of genotype status and could therefore be used in combination with antibiotics as a novel strategy for antibiotic resistance.

## Background

*Helicobacter pylori* is a microaerophilic Gram-negative bacterium which colonizes the human gastric mucosa. It is known to be a gastric pathogen of humans associated with chronic gastritis, peptic ulcers, atrophic gastritis, intestinal metaplasia and lymphoma or cancer development [1,2]. Approximately 50% of the world population is infected with *H. pylori*, with prevalence rates ranging from 20% to more than 80% in certain countries [3]. *H. pylori* has been identified as group 1 carcinogen by the International Agency for Research on Cancer [4]. The observation that only a subset of infected individuals develops severe gastroduodenal diseases may depend on the virulence of the infecting organism. Amongst the different genetic determinants involved in *H. pylori* virulence are the cytotoxin-associated gene (*cagA*) and the vacuolating cytotoxin gene (*vacA*). *VacA,* which is present in all *H. pylori* strains, contains at least two variable parts relevant to virulence [5]. The s region encoding the signal peptide exists as s1 or s2 allelic types, and the m region (middle) occurs as m1 and m2 allelic types [6]. *CagA*, which is not present in every *H. pylori* strain [7], is a marker for a pathogenicity island (PAI) [8] associated with more severe clinical outcomes [9]. It has also been demonstrated that *CagA* is required to disrupt the organization of apical junctions and perturb epithelial differentiation [10]. Type s1/m1 strains produce a higher level of cytotoxin activity than other genotypes. A strong association between *cagA* and *vacA* signal sequence type s1 has been reported [5]. Strains carrying s1 m1 mosaic combination secrete vacuolating cytotoxin in contrast to those with s2 m2 activity [11].

The standard treatment for *H. pylori* related disease is a combination of antimicrobial agents and anti-acid agents [12]. However, side effects for these regimes are common and a major concern is the development of antimicrobial resistance [13]. As a result, several naturally occurring substances have been investigated as potential alternatives for the treatment of *H. pylori* infection [14-18].

Almonds (*Prunus dulcis* D.A. Webb) are a rich source of nutrients and phytochemicals such as vitamin E, monounsatured fatty acids and polyunsatured fatty acids [19]. Other health promoting compounds mainly present in almond skins are polyphenols which have been shown to be bioaccessible during simulated digestion in the gut [20,21]. Among polyphenols, flavonoids are secondary metabolites well documented for their biological effects, including anticancer, antiviral, antimutagenic, anti-inflammatory and antimicrobial activities [22-24]. We have previously demonstrated that polyphenols from almond skins are active against Gram-positive bacteria including *Staphylococcus aureus* and *Listeria monocytogenes* and the Gram-negative *Salmonella enterica*[25]. Natural almond skins also induced a significant decrease in Herpes simplex virus type 2 replication [26]. The antioxidant and anti-inflammatory potential of almond skin polyphenols has also been demonstrated using an experimental model of inflammatory bowel disease [27].

The aim of the present study was to investigate the antimicrobial properties of natural almond skins before and after simulated human digestion in the upper GI tract and the pure flavonoid compounds epicatechin, naringenin and protocatechuic acid against *H. pylori* strains isolated from gastric biopsies of subjects attending an outpatient clinic in Southern Italy. Their clinical relevance has also been elucidated.

## Methods

### Almond skins

Natural almond skins (NS) were prepared from Californian almonds by treatment with liquid nitrogen as previously reported [20].

### In vitro digestion studies

The protocol used to simulate digestion of natural almond skins under gastric and duodenal conditions in vitro has been previously described [21].

Briefly, for the gastric digestion, 1.5 g of NS was suspended in 12.4 mL acidic saline (150 mM NaCl, pH 2.5) and readjusted to pH 2.5 with HCl. Phosphatidylcholine (Lipid Products, UK) vesicle suspension, pepsin (Sigma, UK) and gastric lipase analogue (Amano Enzyme, Japan) were added so that the final concentrations were 2.4 mmol/L, 146 U/mL and 60 U/mL, respectively. Gastric digestion was performed in a shaking incubator (170 rpm, 37°C) for 2 h.

For the simulated gastric plus duodenal digestion, the pH was raised to 6.5 by addition of NaOH and the following enzymes were added: α-chymotrypsin (Sigma, 5.9 U/mL), trypsin (Sigma, 104 U/mL), colipase (Sigma, 3.2 μg/mL), pancreatic lipase (Sigma, 54 U/mL), and α-amylase (Sigma, 25 U/mL) in the presence of sodium taurocholate (4 mmol/L) and sodium glycodeoxycholate (4 mmol/L). Gastric plus duodenal digestion was performed in a shaking incubator (170 rpm, 37°C) for 1 h.

### Almond skin extracts

Polyphenol-rich extracts from NS, NS post in vitro gastric digestion (NS G) and NS post in vitro gastric plus duodenal digestion (NS G + D) were prepared as previously described and their composition has been previously reported [21].

### Patients, *H. pylori* strains and culture conditions

Two reference American Type Culture Collection strains of *H. pylori* (ATCC 43504 and ATCC 49503) and thirty two clinical isolates recovered from gastric biopsy samples of dyspeptic adults (23 women, 9 men; average age, 51 years) undergoing digestive endoscopy at the Endoscopy Unit of the Department of Internal Medicine of the University of Messina, Messina, Italy, were used in this study. None of the patients had previously undergone eradication therapy. All study subjects gave their informed consent and the study was approved by the local ethical committee (Comitato Etico Scientifico A.O.U. Policlinico “G. Martino” Messina, Italy). Diagnosis of peptic ulcer (PU) and non-ulcer dyspepsia (NUD) or gastritis was based on endoscopic examination of the stomach and duodenum. Biopsy samples were taken for each patient for culture. Isolates were derived from patients suffering from gastritis (*n* = 27; 84.37%), or NUD (*n* = 5; 15.62%).

Gastric biopsy specimens for culture were placed in the sterile screw-capped tubes containing 0.5 ml sterile saline and transported to the microbiology laboratory within 2 h. The samples were soaked and sowed in selective (Pylori agar, Bio*Mérieux*) and non-selective (Columbia agar with 7% horse blood, CB, Oxoid) culture media. Cultures were incubated for 7 days at 37°C under microaerophilic conditions. Grown bacteria were identified as *H. pylori* by typical morphology, Gram staining results and positive reactions to oxidase, catalase, and urease activities. The *cagA* and *vacA* status as a virulence factors have been determined in all strains by PCR method.

All strains were harvested by suspension in Brucella broth (Difco) supplemented with 10% fetal bovine serum (BB, Euroclone) and 30% glycerol and stored in liquid nitrogen until used.

### DNA extraction from *H. pylori* isolates

DNA was extracted from *H. pylori* isolates using the QIAamp DNA Mini Kit (Qiagen, Milan, Italy) according to the manufacturer’s instructions. Briefly, one colony was harvested from an agar plate and added to an appropriate volume of phosphate-buffered saline homogenized by vortexing. Twenty microliters of a proteinase K solution (20 mg/mL) and 200 μL of buffer AL provided in the kit were then added, followed by incubation at 56°C for 10 min. Next, 200 μL of ethanol (96%) were added. The mixture was then loaded onto the QIAamp spin column provided in the kit and centrifuged at 6000 g for 1 min. The QIAamp spin column was placed in a 2-mL collection microtube, and the tube containing the mixture was discarded. The column material was washed (500 μL each) with the first washing buffer (buffer AW1) and with the second washing buffer (buffer AW2) provided in the kit. Finally, the DNA was eluted with 150 μL of a third buffer (buffer AE) provided in the kit.

### Oligonucleotide primers

The primers targeting the *vac*A gene (region m and region s) and *cag*A genes [28] used in the PCR assay for the analysis of *H. pylori* isolates, are reported in Table 1. The primers were synthesised by MWG-Biotech AG (Mannheim, Germany).

**Table 1:** **Primers used for cytotoxin-associated gene ( *cagA *) and vacuolating cytotoxin gene ( *vacA *) typing of *H. pylori***

**Gene target**	**Primer designation**	**Nucleotide sequence**	**Amplicon size (bp)**
*vacAS-F*	VacAS-F	5’-ATGGAAATACAACAAACACAC-3’	259 (type s1)
	VacAS-R	5’-CTGCTTGAATGCGCCAAAC-3’	286 (type s2)
*vacA midregion*	VacAM-F	5’-CAATCTGTCCAATCAAGCGAG-3’	567 (type m1)
	VacAM-R	5’-GCGTCAAAATAATTCCAAGG-3’	642 (type m2)
*cagA*	CagA-F	5’-GATAACAGGCAAGCTTTTGAGAGGGA-3’	393
	CagA-R	5’-CCATGAATTTTTGATCCGTTCGG-3’	

### PCR conditions

The amplification was performed using a PCR SprintThermal Cycler (Hybaid, Ashford, UK) and carried out in 50 μL reaction volume containing 200 μmol/L (each) dNTP, 0.1 μmol/L (each) primer, 1X PCR buffer, 50 mmol/L KCl, 10 mmol/L Tris–HCl pH 8.8, 0.1% Triton X-100, 50 mmol/L MgCl_2_, 2 U of Taq DNA polymerase and 5 μL of template DNA or water for the negative control. The temperature profile for the PCR was as follows: an initial step of 4 min at 95°C, followed by a denaturation step for 1 min at 95°C, an annealing step for 1 min at 52°C (for *vacA* PCR) or 59°C (for *cagA* PCR), and a primer extension step for 1 min at 72°C. After the 35th cycle, the extension step was prolonged for 10 min in order to complete synthesis of all strands after which the samples were kept at 4°C until analysis. A negative control lacking of the DNA template was included in each experiment. The *H. pylori* strains used as positive controls in the PCR tests included *H. pylori* ATCC 43504 and *H. pylori* ATCC 49503. Detection of PCR products was performed by gel electrophoresis. Samples (5 μL) of final PCR products were loaded onto 1.5% agarose gel and subjected to electrophoresis in 1X TAE (0.04 mol/L Tris–acetate, 0.001 mol/L EDTA) buffer for 60–90 min at 100 V. The gels were stained with ethidium bromide and photographed under UV light trans-illumination. A 100-bp DNA ladder (BioLab New England, Celbio, Milan, Italy) was included on each gel as a molecular size standard.

### Susceptibility testing

The minimum inhibitory concentration (MIC) was assayed by the standard agar dilution method according to the guidelines of the Clinical and Laboratory Standards Institute (CLSI) [29] using CB. Twofold serial dilutions of the compound tested ranging from 0.016 μg/mL to 1.024 μg/mL were used. Frozen stock cultures were thawed and subcultured on CB and grown for 3 days under microaerophilic conditions. Bacterial growth was taken from the plates, resuspended in BB and grown under shaking (125 rpm) at 37°C for 24 h. *H. pylori* cultures in the exponential phase of growth were diluted with BB to contain about 5 × 10^7^ CFU/mL by adjusting the turbidity of the suspension to match the MacFarland no. 1 standard. Ten-microliter aliquots of the suspension were inoculated on CB containing twofold serial dilutions of the compound tested. Compound-free CB media were included in each experiment to confirm the viability of the inoculum and to observe the growth of any contaminants. CB incorporating twofold serial dilutions of the solvent dimethil sulfoxide was included as a growth control to ensure that the viability of the *H. pylori* strains was not affected by the dimethil sulfoxide used to dissolve the compound. All plates were incubated at 37°C in a microaerophilic atmosphere and examined after 3 days. For quality control, *H. pylori* ATCC strains 43504 and 49503 were tested in each run. Amoxicillin (Sigma Aldrich S.r.l., Italy), and clarithromycin (Abbott S.p.A., Italy), were used as control compounds for comparative analyses. According to CLSI breakpoints, the resistance breakpoints were 0.5 μg/mL for amoxicillin and 1 μg/mL for clarithromycin [29]. The MIC was considered the lowest concentration at which the compound inhibited the development of visible bacterial growth on the agar plates. All MIC determinations were performed in duplicate for each strain.

## Results

To type the *H. pylori* strains isolated from the patients examined in this study, we amplified by PCR different alleles of the genes of the two major virulence factors of this bacteria, *cagA* and *vacA*. The amplification results are shown in Table 2. Fifteen out of 32 *H. pylori* isolates were *cagA* positive, representing 55.5% (15/27) of the isolates recovered from patients with gastritis. No strain identified from patients with NUD was *cagA* positive. The prevalence of the allelic variants of s1 and m1 of *vacA* was higher in the strains isolated from patients with gastritis compared with the strains isolated from NUD patients (77.8% versus 60%, and 63% vs 40%, respectively). When the *cagA* and *vacA* genotypes were combined and analyzed in relation to the clinical outcome (Table 3), the *cagA +* strains with the allelic variant s1m1 of *vacA* were only present in the strains isolated from gastritis patients (53.3%).

**Table 2:** **Prevalence of *cagA *and allelic variants of *vacA *on the *H. pylori *strains**

**Gastroduodenal condition**	***CagA***		***VacA***			
	***CagA *+**	***CagA *-**	**s1**	**s2**	**m1**	**m2**
Gastritis ^*^	**15** (55.5%)	**12** (44.5%)	**21** (77.8%)	**6** (22.2%)	**17** (63%)	**10** (37%)
NUD ^**^	**0** (0%)	**5** (100%)	**3** (60%)	**2** (40%)	**2** (40%)	**3** (60%)

^*^Strains isolated from patients with gastritis (*n* = 27) ^**^Strains isolated from patients with non-ulcer dyspepsia (*n* = 5).

**Table 3:** **Prevalence of *cagA *related to the main allelic combinations of *vacA***

**Gastroduodenal condition**	***CagA*+**			***CagA*-**		
	**s1m1**	**s1m2**	**s2m2**	**s1m1**	**s1m2**	**s2m1 s2m2**
Gastritis^*^	**8**(53.3%)	**5**(33.3%)	**2**(13.4%)	**6**(50%)	**2**(16.7%)	**3**(25%) **1**(8.3%)
NUD^**^	**0**(0%)	**0**(0%)	**0**(0%)	**1**(20%)	**2**(40%)	**1**(20%) **1**(20%)

^*^Strains isolated from patients with gastritis (*n* = 27) ^**^Strains isolated from patients with non-ulcer dyspepsia (*n* = 5).

The MIC values of natural almond skin (NS), NS post in vitro gastric digestion (NS G) and NS post in vitro gastric plus duodenal digestion (NS G + D) against 34 *H. pylori* strains including 2 ATCC *H. pylori* strains are shown in Table 4. Results of negative controls containing DMSO (maximum 1% v/v) indicated the complete absence of inhibition of all the *H. pylori* strains tested (data not shown). All extracts inhibited the growth of both the clinical isolates and the reference strains. As expected, NS was the most effective (MIC range, 64 to 128 μg/mL), followed by NS G (MIC range, 128 to 512 μg/mL) and NS G + D (MIC range, 256 to 512 μg/mL). MIC values of 64, 128 and 256 μg/mL NS, NS G and NS G + D, respectively, inhibited the growth of 50% of the *H. pylori* tested strains. These results clearly confirm that all three polyphenol- rich extracts acted as good growth inhibitors against *H. pylori* with different virulence irrespective of the *cagA* and *vacA* status. In other words, there was no difference in the suppression of growth between the 8 *H. pylori* clinical isolates harboring the *cagA*^+^/v*acA*s1/m1 genotype, including the quality control strains (ATCC 43504 and 49503), and the other *H. pylori* genotypes.

**Table 4:** **Minimum inhibitory concentration of almond skin extracts against *H. pylori *(ATCC strains and clinical isolates)**

	**MIC range**	**MIC **_**50**_	**MIC **_**90**_
NS	64-128	64	128
NS G	128-512	128	256
NS G + D	256-512	256	512

Values are expressed as μg ml^-1^.

NS: Natural almond skin polyphenol-rich extract.

NS G: Natural almond skin polyphenol-rich extract post gastric digestion.

NS G + D: Natural almond skin polyphenol-rich extract post gastric plus duodenal digestion.

The MIC results of epicathechin, naringerin and protocatechuic acid against *H. pylori* strains are reported in Table 5. Protocatechuic acid showed the greatest activity with MIC values of 128 μg/mL and 256 μg/mL against 50% and 90% of the tested strains, respectively. Epicatechin was the least effective compound against *H. pylori* (MIC of 512 μg/mL against 50% of the *H. pylori* strains).

**Table 5:** **Minimum inhibitory concentration of almond skin flavonoids against *H.pylori *(ATCC strains and clinical isolates)**

	**MIC range**	**MIC **_**50**_	**MIC **_**90**_
Epicatechin	128-1024	512	1024
Naringenin	128-1024	256	512
Protocatechuic acid	128-512	128	256

Values are expressed as μg ml^-1^.

All *H. pylori* strains tested were susceptible to amoxicillin (MIC_90_ 0.25 μg/mL; range between 0.016 – 0.25 μg/mL). The MIC_90_ value of clarithromycin against *H. pylori* isolates was 0.5 μg/mL with MIC values ranging between 0.016 and 4 μg/mL. Two (6%) out of 32 isolates tested were clarithromycin resistant, one of which was isolated from patients suffering from gastritis harbouring the *cagA*^+^/v*acA*s1/m1 genotype.

The two clarithromycin-resistant strains were inhibited by almond skin extracts (NS, NS G, NS G + D) at 128 μg/mL; the MIC values of pure compounds (epicatechin, naringenin, protocatechuic acid) against these two strains were 256, 256, and 128 μg/mL, respectively.

Quality control MICs were within acceptable limits for all antimicrobial susceptibility testing.

## Discussion

The results reported in the present paper demonstrated that polyphenols present in almond skins are effective against *H. pylori* strains, both ATCC and clinical isolates. As previously reported [21,26], NS was the most active against the tested strains. This result could be due to the highest polyphenols concentration in NS, whereas a decrease in the total phenolic content was observed post in vitro gastric and post in vitro gastric plus duodenal digestion [21]. Catechin, epicatechin, kaempferol (aglycone and conjugated) and isorhamnetin (aglycone and conjugated) were the major compounds identified in NS [21], leading to assume the combination of these polyphenols was responsible for the higher activity against *H. pylori*. Quercetin and kaempferol were shown to be active against a *CagA* + and a *CagA*- strain of *H. pylori* and a relationship between antimicrobial potential and antioxidant activity was only reported for the *CagA*- G 21 strain [18]. The same authors have also recently reported an increased susceptibility to resveratrol of *H. pylori* strains isolated from patients suffering from gastric carcinomas [30]. The investigation of the isolated compounds in the present work demonstrated that protocatechuic acid was more active than naringenin and epicatechin and the effectiveness of protocathechic acid against *H. pylori* in broth and stomach homogenates from mice has also been demonstrated by Liu et al. [31], with no differences between antibiotic susceptible and resistant strains. Other investigations have reported promising effect of natural compounds, such as hydrolysable tannins and lignans, on the proliferation of *H. pylori* and the prevention of gastric carcinogenesis [32,33]. Reports on the mechanism of action of a range of flavonoids have shown that isoflavones and chalcones inhibited the urease secreted by *H. pylori* to survive the acidic conditions found in the stomach [34,35]. Other flavonoids may also be responsible for the neutralization of the vacA via interference of the toll-like receptor 4 signaling induced by *H. pylori*[36,37]. A recent study reported that the antimicrobial potential of the oligopeptide C_12_K-2 against *H. pylori* has a dual mode of action on both membrane and cytoplasmatic components [38]. Although the rate of resistance to clarithromycin has significantly increased in several countries (13), the observed resistance to this antibiotic in the *H. pylori* isolates tested in the present work was surprisingly low (6%).

## Conclusions

In conclusion, we have shown that polyphenols from almond skins were effective in vitro against *H. pylori*, irrespective of the bacterial genotype which is independent of the presence of the *cagA*, and could therefore be used in combination with antibiotics as a novel strategy for antibiotic resistance.

## Competing interests

The authors have received a research grant from the Almond Board of California.

## Authors’ contribution

CB, MTF, GM conceived the study and participated in its design. EL, AF, SZ carried out the experiments and performed the data analyses. EL and SZ participated in the isolation of clinical strains. EL carried out the PCR amplification. GM coordinated, supervised the study and critically revised the manuscript. CB, AF, EL, SZ, MTF, GM drafted the manuscript. All authors have read and approved the final manuscript.
